# Community perceptions of mass screening and treatment for malaria in Siaya County, western Kenya

**DOI:** 10.1186/s12936-016-1123-y

**Published:** 2016-02-06

**Authors:** Kathryn Shuford, Florence Were, Norbert Awino, Aaron Samuels, Peter Ouma, Simon Kariuki, Meghna Desai, Denise Roth Allen

**Affiliations:** Centers for Disease Control and Prevention (CDC), Atlanta, GA USA; Kenya Medical Research Institute (KEMRI), Kisian, Kenya

**Keywords:** Mass screen and treat, Malaria elimination, Acceptability, Adherence, Perceptions, Community sensitization, Qualitative methods

## Abstract

**Background:**

Intermittent mass screening and treatment (iMSaT) is currently being evaluated as a possible additional tool for malaria control and prevention in western Kenya. The literature identifying success and/or barriers to drug trial compliance and acceptability on malaria treatment and control interventions is considerable, especially as it relates to specific target groups, such as school-aged children and pregnant women, but there is a lack of such studies for mass screening and treatment and mass drug administration in the general population.

**Methods:**

A qualitative study was conducted to explore community perceptions of the iMSaT intervention, and specifically of testing and treatment in the absence of symptoms, before and after implementation in order to identify aspects of iMSaT that should be improved in future rounds. Two rounds of qualitative data collection were completed in six randomly selected study communities: a total of 36 focus group discussions (FGDs) with men, women, and opinion leaders, and 12 individual or small group interviews with community health workers. All interviews were conducted in the local dialect Dholuo, digitally recorded, and transcribed into English. English transcripts were imported into the qualitative software programme NVivo8 for content analysis.

**Results:**

There were mixed opinions of the intervention. In the pre-implementation round, respondents were generally positive and willing to participate in the upcoming study. However, there were concerns about testing in the absence of symptoms including fear of covert HIV testing and issues around blood sampling. There were fewer concerns about treatment, mostly because of the simpler dosing regimen of the study drug (dihydroartemisinin–piperaquine) compared to the current first-line treatment (artemether–lumefantrine). After the first implementation round, there was a clear shift in perceptions with less common concerns overall, although some of the same issues around testing and general misconceptions about research remained.

**Conclusions:**

Although iMSaT was generally accepted throughout the community, proper sensitization activities—and arguably, a more long-term approach to community engagement—are necessary for dispelling fears, clarifying misconceptions, and educating communities on the consequences of asymptomatic malaria.

## Background

In recent years, much progress has been made in the fight against malaria, resulting in substantial global reductions in mortality and incidence rates—a result of increased funding and commitment to prevention and treatment strategies [1, 2]. However, malaria remains a disease of public health significance around the world, as there were an estimated 214 million malaria cases and 438,000 deaths attributed to it in 2015 [1]. Further interventions are necessary to sustain the progress that has been made and to accelerate the reduction in disease burden, transitioning from strategies of control to those of elimination.

There is increasing recognition of the complexities that come with implementing community wide health interventions like intermittent mass screening and treatment (iMSaT), mass drug administration (MDA), insecticide-treated bed net (ITN) distribution, indoor residual spraying (IRS), and intermittent preventive treatment (IPT), which are aimed at malaria control and prevention [3–7]. Okello and colleagues [4] comment on the results from past studies on the acceptability of such interventions which reveal a variety of influencing factors including perceptions of disease burden and aetiology; perceptions of the safety, effectiveness, and benefits of the treatment or intervention; individual, social and cultural factors within the community; and structural and system factors. This body of literature covers a range of interventions and tools including IPT [6–9], iMSaT [10], RDTs [11, 12], IRS [13], and community case management of malaria and seasonal malaria chemoprevention [14]. The literature on the acceptability of iMSaT in the general population is minimal but includes a recent paper from a study conducted in Zambia [3]. Researchers discuss various testing-related fears and misconceptions as well as inadequate information about the study as primary reasons for refusals. They underscore the importance of increased community sensitization and improved communication to counter misinformation and to increase acceptance and effectiveness of the intervention. It is essential to understand community perceptions both before the intervention in order to plan its delivery and ensure optimum uptake [15–17] as well as after the completion of treatment rounds so as to improve effectiveness and strategies for scale-up [3].

The Centers for Disease Control and Prevention (CDC) and the Kenya Medical Research Institute (KEMRI) have been collaborating on malaria research and public health programmes in western Kenya for over 35 years. Researchers are currently evaluating iMSaT as a possible additional tool for malaria control and prevention in Siaya County, western Kenya. The iMSaT study will assess the impact of repeated rounds of mass screening and treatment on malaria transmission, morbidity and mortality over a 2–3 year period. In this strategy, every individual within a community is screened for malaria with a rapid diagnostic test (RDT) at the household level, regardless of the presence or absence of symptoms, and persons found to be positive are treated with dihydroartemisinin-piperaquine (*Duo*-*Cotecxin*^*®*^, Holley-Cotec Pharmaceuticals Co. Ltd., Beijing), taken once a day for a total of 3 days. Nested within the iMSaT study is a qualitative data collection component to document community perceptions on the iMSaT intervention (including testing and treating) before and after the first implementation round. This paper will present the findings from this latter component.

## Methods

### Study site

The study was conducted in Siaya County in Nyanza Province in western Kenya—an area of high malaria transmission throughout the year, where the prevalence of malaria parasitaemia is approximately 40 % in the general population (unpublished data, KEMRI-CDC). Residents are primarily from the Luo ethnic group, earning a living through subsistence farming, fishing, and small businesses [18]. In 2001, KEMRI-CDC launched a health and demographic surveillance system (HDSS) in Nyanza Province. The KEMRI-CDC HDSS conducts malariometric monitoring through a variety of methods, including hospital and outpatient surveillance, annual parasitaemia and anaemia surveys, monthly entomological surveys, and entomological insecticide resistance testing, among others. The communities living in the study area are, therefore, familiar with KEMRI-CDC’s work, often participating in clinical trials and other epidemiological studies over the past few decades. Malaria transmission in the area is high and perennial, with peak transmission in May–July and October–November. *Plasmodium falciparum* is the dominant malaria parasite species. In July 2006, artemether–lumefantrine (*Coartem*^*®*^, Novartis Pharmaceuticals Corporation, Basel) was introduced as first-line treatment for uncomplicated malaria and was provided in government and mission health facilities; in 2012, the Kenya Ministry of Health adopted a universal policy requiring diagnostic confirmation of all individuals with reported or documented fever where this is possible before they are given treatment for malaria in facilities where this is possible [19]. Siaya County also bears one of the highest HIV prevalence rates among adults (17.8 %) in the country, following Homa Bay (27.1 %), and Kisumu (18.7 %). These most recent figures from 2012 compare with a national average prevalence of 5.6 % among adults aged 15–49 years [20].

### Qualitative study design

The overall aim of the iMSaT qualitative data collection component was to explore community perceptions of the screening and treatment intervention before and after the first implementation round in order to identify aspects of the iMSaT activities that might be improved in future rounds or if iMSaT becomes policy. The pre-implementation round was intended to serve as a reference point and to provide sociocultural information that might be relevant for the initial round of iMSaT activities. The purpose of the post-implementation data was to provide community feedback on the first round of iMSaT to identify possible areas where iMSaT procedures should be strengthened or revised or whether additional community sensitization was needed. Topics included general information about community knowledge of malaria, malaria care-seeking and prevention behaviours, and preferences of anti-malarial drugs, but focused primarily on perceptions of and experiences with malaria testing and treatment in the absence of symptoms in order to anticipate reasons for refusal to participate in the study.

### Selection of study communities

Six study communities were randomly selected in the KEMRI-CDC HDSS area (two each from Karemo, Gem, and Asembo which were all administrative districts at the time). These villages formed part of the total 41 intervention communities of the wider iMSaT study. Qualitative data were collected in the same six communities twice: once in July 2013 (pre-implementation), and again in November 2013 (post-implementation). The timing of these data collection rounds corresponds to the periods of peak malaria transmission in Kenya (May–Jul; Oct–Nov). This paper presents only the data before and after the first round of iMSaT in order to capture initial perceptions and describe their role in informing the first rounds of iMSaT delivery. Because the iMSaT intervention involves multiple rounds, it is reasonable to expect that community perceptions after the first round might evolve after several repeated rounds; for this reason, an additional set of qualitative data was collected. Analysis is underway and will be reported separately.

### Data collection methods and participant recruitment

Qualitative data were collected by an eleven-member field team consisting of a field supervisor, research assistant, and nine interviewers. Additionally, the co-investigator for the qualitative component conducted the interviewer training and oversaw data collection for the pre-implementation round. Data collection methods included focus group discussions (FGDs) with community members, and small group or individual interviews with community health workers (CHWs) in each study community, depending on the number of CHWs available. All interviews were conducted in the local dialect Dholuo, digitally recorded, and transcribed into English within 48 h of completion. A total of 36 FGDs and 12 interviews with CHWs were conducted.

FGDs were used to explore community experiences and perceptions of malaria testing and treatment in the absence of symptoms. Other main topics included community knowledge about malaria etiology, prevention, and treatment, malaria care-seeking and prevention behaviours, and knowledge and opinions of anti-malarial drugs.

Three FGDs were conducted in each of six study communities per data collection round: one with community opinion leaders, one with adult males, and one with adult females. Each FGD consisted of 5–12 participants. Participants for the opinion leader FGDs were purposively selected in consultation with community leaders and sometimes included both men and women. Participants for the male and female FGDs were randomly selected from a list of village members from the HDSS, which was then provided to village leaders in advance of interviews. Village leaders visited consecutive names on the list until 12 community members agreed to participate in the FGD. Occasionally, a community member from the original list of 12 did not turn up for the FGD (or the village leader failed to inform them in advance); in this case, the village leader appointed another community member to fill his/her place.

The CHW interviews explored community perceptions of malaria testing and treatment in the absence of symptoms as well as malaria caseload and management, and the CHWs’ role in the iMSaT activities. All CHWs in the study community were invited to take part in the CHW interviews. Although the original study design included FGDs with CHWs, the number of CHWs residing in the study communities ranged from one to five. As a result, the format of the CHW interviews depended on the number of available CHWs. Small group CHW interviews were conducted in all six communities during the pre-implementation round and in five of six study communities during post-implementation; an individual CHW interview was conducted in the remaining community where only one CHW was available (yielding a total of 12 CHW interviews from both rounds). Total numbers of participants by FGD/interview type in each round are listed in Table 1.

**Table 1:** Total number of participants by interview type and data collection round

FGD/Interview type	Pre-iMSaT round	Post-iMSaT round
Opinion leader	52	52
Male	41	51
Female	52	49
CHW	17	20

### Interview procedures

Before the start of qualitative study, an initial round of sensitization meetings had already been conducted among communities in the study area in order to introduce the iMSaT intervention and explain the purpose of treating asymptomatic malaria. During the first round of interviews (pre-implementation), the iMSaT study was again introduced, and participants were shown a sample RDT kit (Carestart™ Malaria HRP-2) as well as a sample of the study drug (Duo-Cotecxin^®^). This was to facilitate discussion and to assess their willingness to undergo testing and treatment as part of the upcoming intervention. The discussion focused on perceptions of Duo-Cotecxin as it was the study drug used in the first two rounds of iMSaT; however, a different brand, Eurartesim^®^ (Sigma-Tau Pharmaceuticals Inc., Pomezia), was used in subsequent rounds. The moderator explained that the drug was to be taken once a day for a total of 3 days, unlike the familiar malaria drug available in the community (Coartem^®^), which requires multiple doses throughout the day. Participants were allowed to touch the RDT kit and the drug packaging, and any questions concerning the drug were addressed. They were also informed that the drug would offer them protection from malaria for a period of 6 weeks unlike Coartem, which would offer a protection period of 2 weeks. Respondents were asked their opinion of being tested and/or treated in the absence of symptoms.

### Data management and analysis

Digitally recorded interviews were transcribed directly into English from Dholuo. Upon completion, all transcripts were reviewed for accuracy by FA, a native Dholuo speaker. No personal identifiers were used in the transcripts. The transcripts were then imported into NVivo8 for coding and content analysis. During the coding process, each transcript was read twice, line-by-line, and thematic codes were applied to relevant portions of text. The initial coding template was developed by DRA and KS, and subsequently revised by KS. KS had primary responsibility for coding and content analysis.

### Ethical approval and consent procedures

Ethical approval for the study was obtained from KEMRI’s Ethical Review Committee (reference #SSC 2380) and CDC’s Institutional Review Board (reference #6374). Overall community consent was obtained through meetings with community leaders in which a series of presentations were made to explain the purpose of the study. Each community was visited prior to the start of data collection in order to meet with village elders and to collect community descriptors. Written consent (signed or thumb print) was obtained from each FGD and CHW participant prior to beginning the interview process. During both community sensitization and the FGD/interview consent process, community members were reminded of their right to opt out of the study without any consequence to themselves or their families.

## Results

Several overarching themes emerged from the data: general perceptions of research and of KEMRI-CDC; perceptions and experiences of RDTs and testing in the absence of symptoms; perceived purpose and benefit of iMSaT; perceptions and experiences of malaria treatment in the absence of symptoms; concerns about drug availability and affordability, issues around drug adherence, and community suggestions of further sensitization for future iMSaT rounds.

### Pre-implementation results

#### General perceptions of research and of KEMRI-CDC

Although not the primary focus of this study, participants often discussed their perception of research as a whole and their previous experiences with other studies. These comments were often probed with follow-up questions when the content seemed relevant to iMSaT. As noted above, the iMSaT study is being conducted in a part of western Kenya where there is a long and well-established research collaboration between KEMRI and CDC. Participants often invoked this research collaboration in their responses, sometimes referring to it as “KEMRI-CDC”, “CDC-KEMRI”, or other times simply as “KEMRI” or “CDC.” Participants’ views on the role of these research institutions varied. While some expressed a positive view of KEMRI-CDC and were quick to express their appreciation and acceptance of research activities (and specifically of the iMSaT study), others held more negative views. Some of the latter group suggested that participating in such studies was a waste of time, especially when they receive no benefits from participating, and linked this with deeper issues of poverty.

#### Perceptions of RDTs and testing in the absence of symptoms

Respondents were asked about their previous experiences with malaria RDTs and shown a sample testing kit. Generally, among the community members who were familiar with the RDT, most believed it to be a valuable tool and had high expectations of the RDT. They felt that it should be able to detect any illness, not just malaria. The quotations below refer to participants’ previous experiences with RDT testing outside of iMSaT activities.  

*M: What is your thought about this RDT testing [for malaria]? What do you think about it?*

*P4: It’s good because it immediately gives out the result. That’s one of its advantages that I saw; it gives a genuine result.* (Male FGD, Asembo)

*I think it’s good for our life as it gives results very fast whether there’s electricity or not. It will just give results of what you want tested.* (Female FGD, Karemo).

Nevertheless, some who had never been tested with an RDT (and even for some who said they had) associated it with the HIV testing kit and thus believed the RDT could be used for diagnosing both malaria and HIV.  

M: *KEMRI*-*CDC is planning a study where they will visit people in their homesteads to test them for malaria even if they do not have any malaria symptoms…They will use a type of malaria test that provides results within 20* *min* [showing the RDT].

*P6: Yes I have seen this, isn’t it used for testing blood?*

*P3: Yes… it’s used for testing blood…*

*P4: Isn’t it used for the HIV test?*

*M: This is only for malaria.*

*P4: But it looks like the other one*. (Male FGD, Asembo)

When discussing whether the community would accept being tested with an RDT in the upcoming intervention, this association of the RDT with HIV testing led some community members to suspect that iMSaT staff might pretend to test for malaria but actually test for HIV.

*Many people will have different opinions especially now that we have many HIV cases, they will just say that the CDC are pretending but they are testing for HIV.* (Female FGD, Asembo)

*Some will think that they are going to be tested for HIV instead of malaria.* (Female FGD, Karemo)

Additional concerns about testing centered on the issue of giving blood. Many respondents believed that too much blood is taken when they participate in clinical studies while others do not like having their finger pricked. Others expressed concerns about where the blood samples are taken and what they are used for.

*You know that when you come in the name of CDC, like even some of us whose children were taken by CDC, it’s what will make people refuse to participate in this study. When they hear of blood samples [being] taken by the CDC group, they will tell you that they take too much blood in a full syringe like that of a cow, but another person will say that a little blood sample is taken for test. That is what will make people refuse to participate in the study because CDC has a bad reputation.* (Female FGD, Karemo)

*Majority say that they remove lots of blood from people and take it somewhere and when a child dies they remove the body parts. And as a result people get scared about CDC tests but I welcome it because I have found help from it.* (Female FGD, Asembo)

#### Perceived purpose and benefit of iMSaT

There was a wide range of understanding regarding the purpose and benefit of the iMSaT study. Although the concept of asymptomatic malaria infections and the purpose of treating them was explained during initial sensitization meetings, not all of those interviewed recognized the value of testing in the absence of malaria symptoms. This lack of understanding of asymptomatic malaria led to a variety of concerns with both testing and treatment.  

*M: In your opinion, how do you think people will feel about being tested for malaria with an RDT when they do not feel sick?*

P1:*From my experience I cannot accept to be tested when I don’t feel sick…I will wait until I feel sick to be tested.* (Opinion leader FGD, Asembo)  

*M: How do you think people will feel about malaria testing when they do not have any symptoms?*

*P11: On this people’s perceptions will differ, some may send them away, those with knowledge who have heard of this will accept; therefore people’s perceptions will differ [depending] on how knowledgeable they are.* (Opinion leader FGD, Karemo)

There were some participants, however, who communicated an awareness of asymptomatic malaria—some more clearly than others—and thus recognized more readily the value in testing and the purpose of iMSaT. Respondents used a variety of phrases and ideas to convey this, such as the concept of “hidden malaria.”

*There [are times when] you may feel that you don’t have malaria, but you have hidden malaria, therefore it is only a test that can show whether you have malaria.* (Male FGD, Gem)

*Someone can live with malaria in their bodies for long without knowing it…you think you don’t have malaria but it’s already in you, so if you get an opportunity to be tested before the malaria symptoms show in your body then that will be a good opportunity.* (Female FGD, Asembo)

#### Perceptions about malaria treated in the absence of symptoms

Compared to concerns about testing in the absence of symptoms, there was less concern expressed about receiving treatment in the absence of symptoms (once tested). Most participants felt that if the test showed they were positive for malaria, they would welcome the treatment without hesitation. When participants were asked about their perception of the study drug Duo-Cotecxin, some drew on their previous experiences with the drug which had been introduced fairly recently in some clinics but was not yet widely accessible. The majority of responses about Duo-Cotecxin were very positive—mostly due to its perceived high effectiveness and simpler dosing regimen as compared with the current first-line drug Coartem, which people perceived as having lost its “power” and being quite burdensome in terms of dosage.

*It will be easy for them for now…Coartem and the other one, even when taken for days, it’s not effective. You take and after 1* *week or 2* *weeks the malaria is still the same.* (Opinion leader FGD, Gem)

*It is less, so you find that with [Coartem], one gets tired with taking it on the way and abandons it because you take four tablets and two tablets of Panadol, so it’s not easy…we can encourage them that this new drug is easier.* (CHW FGD, Asembo)

#### Drug adherence issues

This discussion of adherence issues emerged as a central theme as many participants highlighted the importance of completing the full treatment dose although admitted they often fail to do so.

*Depending on the doctor’s prescription, even when feeling well, it is better for a patient to continue taking or finishing the dosage. That is why it is not possible to cross a river without passing through water.* (Opinion leader FGD, Asembo)

*I notice some change then I know the medication I was given has helped me, but if I don’t notice any change it could be that I got lazy and did not finish my dose and so the sickness persists, so I just take it for granted that the medicine was not effective but I am the one who did not complete the dosage.* (Female FGD, Asembo)

Other reasons for not completing the full dose may be due to adverse side effects or wanting to save partial doses for when they are ill again.

*In our community people like dividing drugs to others, somebody takes half and the other people also take half dose. Therefore with us we may see that the drug is efficient, so if somebody is given drugs, he should not share until the dose is over so that habit should stop.* (Male FGD, Gem)

#### Other drug-related concerns

Although the majority of comments surrounding Duo-Cotecxin were positive, some participants cited ‘fear of new things’ as a potential reason for refusal to take the drug.  

*M: What are the possible reasons for refusals?*

*P2: Some people fear…such that anytime new things are done they are just suspicious. That could be a reason for refusal…*

*P4: …you do not want to change your mind, ideas of the past, that’s why people fear*

*P5: People always fear new things. For some people think…‘These new things, do they want to spoil us or do they want to make us good’?* (Opinion leader FGD, Gem).

An additional concern included the potential side effects of Duo-Cotecxin, but some participants suggested that most community members would comply with the treatment as long as it proved effective and did not cause too many side effects.

Participants also wondered about the affordability and availability of Duo-Cotecxin based on their previous experience with the high cost and/or limited drug supplies.

*The concern they may have is, in case the price can go down, that it be locally available, not only at [name of local private health center]. It should be looked into that the pill becomes available in government hospitals so as to reach the common man.* (Opinion leader FGD, Karemo)

#### Community suggestions for iMSaT sensitization activities

Participants were given the opportunity to provide suggestions on what should be done to encourage people to participate in the study and to address concerns with testing and treatment. There were many recommendations for further sensitization in the communities in order to raise awareness and clarify any misconceptions about the purpose of the study. While some participants recommended individual counseling during the screening and treatment process, others felt that schools and churches were the ideal pathways for sharing information.

*It depends on someone’s understanding. It is a good idea for a person who is doing the testing to talk or counsel the person tested for malaria to understand the results that show he is positive for malaria but still they do not feel sick.* (Opinion leader FGD, Asembo)

*What we think is this: if you want to come for such occasions, tell us in advance. Just go to the school…when it is announced in schools, our children are there, and they will then tell us that the malaria team will come tomorrow. It is easily done through schools. We get information from schools through teachers…the only way to help us is to send messages through the teacher…or through the church, the church on Sunday.* (Opinion leader FGD, Gem)

Many participants, especially opinion leaders, believed they could also play a role in sensitization by talking with community members and sharing the information they had learned during the focus groups.

*We should encourage each other by involving one or two people who have been trained here in [village] to educate more people…on what the exercise of mass screening is going to entail. Then educating more people on what the testing and treatment of malaria is all about.* (Female FGD, Karemo)

#### iMSaT sensitization activities

After the completion of the pre-implementation round, preliminary results with respect to community suggestions about the need for sensitization were promptly shared with the wider iMSaT study team. Prior to implementation of iMSaT, a round of sensitization activities was conducted in study villages targeting the local community, opinion leaders, community advisory board (CAB) members and CHWs with the aim of addressing study concerns. The iMSaT community sensitization team visited communities to explain how the study fits within the agenda of malaria control, introduced thematic areas of the study (such as the concept of asymptomatic malaria), and discussed basic procedures of participation/selection and potential benefits of participation.

### Post-implementation round

#### Overall perceptions of iMSaT

Findings from the qualitative interviews conducted after the first round of iMSaT was completed indicate that many participants were positive about their overall experiences during the iMSaT household visits and expressed their appreciation and willingness to continue with the study. Compared to the pre-iMSaT round, the comments during post-iMSaT interviews also tended to be more positive about KEMRI-CDC.  

*M: What are people in this community saying about the iMSaT study?*

*P3: We as those people who were tested are really positive*

*P8: After doing the rounds, people from this community appreciated the activities of the iMSaT study*

*P5: I have heard most people talking positive about the IMSAT activities.* (Male FGD, Asembo)  

*P5: What I hear people saying is that the study has really helped people. Even those who didn’t feel sick were found with malaria and were given drugs. So they appreciated that the study was good*

*P1: That’s what I heard…people appreciating, and I also appreciated it.* (Female FGD, Gem)

Some respondents emphasized the importance of the study team’s attitude and their exchange with community members during household visits. They also highlighted the perseverance of the iMSaT teams as they returned to households in attempts to screen missed groups.

*The team worked very well. In fact, there are places that they could go to and find children playing outside; they would play with those children first to calm them down owing to the fact that children are scared of being injected/pricked…so they would play with those kids, soothe them until they accepted. The team worked very well. They were humble and walked in peace.* (Opinion leader FGD, Asembo)

*P4: They were very humble people…aah! Because when they came to my house, I was in the garden but they took their time…then they tested me, they were so humble and patient people.*

*P2: I loved them because when they came, they could teach first what they were to do and only when you agreed with their programme is when they would go ahead with their testing and treatment. *(*CHW FGD, Karemo, speaking from perspective of study participants, as they were not part of iMSaT staff*)

#### Experiences with malaria testing

Despite the overall positive comments about the iMSaT intervention, concerns about malaria testing remained. Many participants still expressed concerns about being tested for HIV or had heard rumours that the study teams were using malaria as a ploy and actually screening for HIV.

*The challenge I encountered though small was that some people would accept to be tested and others wouldn’t. The ones who would refuse would ask questions such as ‘this test you’re doing isn’t for malaria. You’re lying to us that this test you’re doing is for malaria, isn’t it?’* (CHW FGD, Asembo)

*Most people in this community fear the tests as they always think that every test being conducted is for HIV/AIDS…so that’s why you see people running away because they fear the tests.* (Male FGD, Karemo)

The issues around blood samples—both fear of giving blood and confusion as to where the samples were taken—again proved to be a main source of unease or dissatisfaction with the process, and oftentimes, a reason for refusal to participate in the study.

*Some people feared that they have little blood and [said] the iMSaT teams were like blood suckers.* (Female FGD, Gem)

*The reason some refused was that most people, even those who live in this village, think the KEMRI people want to sell their blood.* (CHW FGD, Asembo)

During household visits, a finger prick blood sample was also collected for preparation of dried blood spots on filter paper for assessment of RDT sensitivity. Respondents were particularly confused about this use of filter paper in addition to RDTs. It is unclear whether this confusion was due to their own misunderstanding or rather to lack of or misinformation given by the iMSaT teams.

*After testing me, my blood sample was taken on the piece of paper for further screening. It’s not easy for me to get the result…this is not easy for us to understand…why the result cannot be given back to me, to know what is going on or what was found wrong in me.* (Male FGD, Asembo)

*I can remember being told that it would be taken to Atlanta, somewhere in Atlanta but the details I’ve forgotten when they said it would be back but remember being told it would be taken to Atlanta somewhere.* (Opinion leader FGD, Karemo)

#### Experiences with malaria treatment

As anticipated from the pre-implementation round data, there were fewer concerns with the treatment component of iMSaT, and it was rarely cited as a reason for refusal. Many participants expressed their appreciation for the treatment and praised the drug’s effectiveness.

It was discovered that some community members (from all three districts) had adapted the name of the study drug from Duo-Cotecxin to ‘*Duokoteko*’ meaning ‘giving energy’ in the local language.

*What I saw interesting during the visit was…when a person who was screened and found to be malaria positive, then Duo*-*Cotecxin was given, and she got healed and felt much better so from that time she started calling Duo*-*Cotecxin drug ‘Duokoteko’* [laughter]. (CHW FGD, Asembo)

*No, we have no problem because it is the same as…it’s like you are saying some Luo word…‘Duokoteko,’ therefore you know it’s like it reduces pain* [laughter]. (Opinion leader FGD, Karemo)

*M: And what’s that, ‘Duokoteko’?*

*P2: There are some people who had malaria…and after being tested they were given drugs which made them have more energy, that’s why they called it ‘Duokoteko.’* (CHW FGD, Gem)

There were some participants, however, who believed they were being used as “guinea pigs” to test the effectiveness of Duo-Cotecxin.

*It’s like we are being used as guinea pigs to conduct study trials with, yet there is nothing that we are given. Personally, I am not happy with the study activities because we are not gaining anything at all.* (Male FGD, Karemo)

M: *Why did these people refuse to participate in this study?*

*P2: Some people tend to think too far when help comes. They think that the CDC people conduct research on them when they discover a new drug abroad then they come to test it on them to find out if it’s effective. So the community members say they are not monkeys for research to be conducted on them…some people touch on that first then they wait to hear from others about the effects.* (CHW FGD, Asembo)

A common sentiment among those who tested negative was disappointment they had not received any drugs.

*My perception was, I thought after screening I could be given some medicine, even if I was not malaria positive. I could be given some medicine for emergency in case I fall sick but I did not see [it].* (Male FGD, Gem)

Conversely, among those who tested positive and were given treatment, it seems that some may have failed to finish the full treatment dose.  

*M: I would also like to ask you a question, with those who took the medicine, how were they…and even today?*

*P2: I would say that most of those people did not finish the dose.* (CHW FGD, Karemo)

*P4: I have never been at ease with these new drugs because they could have adverse effects that come later*

*M: So, haven’t you taken the drugs you were given?*

*P4: I haven’t taken them* [laughter]. *They do have adverse effects… it’s something still under trial. Those praising the drug may come to cry later.* (Male FGD, Karemo)

#### Respondents’ suggestions for further iMSaT rounds

Respondents again encouraged further sensitization as well as follow-up visits so as to improve participation in subsequent iMSaT rounds. Many respondents commented on the poor sensitization leading up to the study, or lack thereof, and believed it to be the main source of participant refusals.

*This was due to the fact that they were never sensitized…Because all this work was left to the CHWs, and no village elders were selected to help in the mobilization on the impending visit that was to come…So you find that the CHWs come with some bag and explain to you what is going to happen, and since proper sensitization was not done, some people stayed away.* (Male FGD, Gem)

*I would respond by saying that some people refused because of lack of knowledge. Some people would refuse because they don’t know the benefits of what has been brought to them and its goodness. So my advice is before [iMSaT] round 2 begins, mobilization and sensitization should be done in the community. The community members should be able to speak for themselves and say that when the CDC people brought us treatment for malaria, I felt this way after taking the medicine.* (CHW FGD, Karemo)

Some noted that with more sensitization, people would change their mind about participating in the study after seeing positive results from the first round of treatment. However, it is important to note that there were refusals even among those who received sensitization before the study.  

*M: For the people who refused…didn’t you carry out sensitization?*

*P2: Sensitization was carried out, but it is difficult for people to understand things. Some people would accept and even sign the consent form. Just before you begin your work they say, ‘No, test these children and leave me. She is cheating me and what I’m seeing here resembles the HIV test kits. You want to test for HIV, so just test the children and leave me alone.’ So there’s nothing you can do*- *you just test the children and leave her alone.* (CHW FGD, Gem)

Data from the iMSaT study indicated that refusal rates were relatively low, although the number of refusals nearly doubled in Round 2 of iMSaT. A 3.3 % refusal rate was recorded in the first round of iMSaT with a total of 23,199 people sampled and 755 refusals. Round 2 and Round 3 of iMSaT (carried out after the completion of the qualitative study) resulted in refusal rates of 6.0 % (1414/23,699) and 5.6 % (1383/24,676) respectively.

Overall, there was little relevant variation across and within groups: although female respondents seemed to express slightly more concern regarding testing and treatment as compared to their male counterparts, they also tended to share more positive statements about the study (as compared to males). As might be expected given their role within the community, opinion leaders tended to be more outspoken and more readily expressed criticism of KEMRI-CDC and research as compared to other respondent groups. When CHWs were asked to discuss community perceptions and opinions around testing and treatment, their comments tended to align with those of the community members themselves.

## Discussion

Qualitative interviews with community members and opinion leaders revealed mixed opinions of iMSaT activities. In the pre-implementation round, respondents were generally positive and willing to participate in the upcoming iMSaT study. There were, however, some concerns related to testing in the absence of symptoms including fear of being tested for HIV and issues around blood samples. There were fewer concerns about the study drug Duo-Cotecxin although these included affordability and future availability, but most respondents were positive about the treatment due to its simpler dosing regimen. The pre-implementation data were primarily based on hypothetical scenarios or experiences outside the iMSaT study (in order to foresee any major problems with the intervention); however, these issues were largely confirmed in the post-implementation data. After the first round of iMSaT, there were less concerns and negative views expressed, although some of the same issues around testing remained, as these were the most common reasons cited for refusal. Although not the primary focus of this study, community perceptions of the KEMRI-CDC research and public health collaboration cannot be divorced from the perceptions of the iMSaT study. The results suggest that participants’ views and acceptance of iMSaT activities were heavily influenced by their perception of research as a whole and their previous experiences with other studies.

### Barriers to acceptability: rumours and misconceptions

The majority of concerns during both pre- and post-iMSaT centered on fear of covert HIV testing and issues related to having samples of blood taken (e.g., confusion surrounding the purpose of the filter paper samples which were used to test RDT sensitivity). These findings are consistent with both Silumbe et al. [3] and Okello et al. [4], which serve as the most directly relevant publications on the subject thus far—providing a set of pertinent and overlapping themes.

Rumours about blood (blood-stealing/selling/trading, deliberate spread of disease) are quite common and widespread across sub-Saharan Africa [6, 21, 22] and have been reported from the region since colonial times [21–23]. They can affect recruitment of study participants, withdrawals/refusals, or adherence issues, but other times have no direct impact on research [3, 4, 17, 24]. The medical research community has tended to interpret such rumours and misconceptions as expressions of ignorance of medical science and the research process, or as adherence to traditional beliefs. Findings from some studies, however, indicate that rumours should not be ignored, given their potential effect on research and public health interventions; addressing the rumours and engaging with communities could improve relations and understanding between researchers or public health institutions and study participants [21, 22]. For this reason, and considering the small yet increasing refusal rates for iMSaT rounds 2 and 3, a subsequent round of qualitative data has been collected to explore the potential causes. These data are currently being analysed.

### Issues of adherence

Among community members, it was clear that knowledge of malaria was high, as was their awareness of the burden and consequences, but there was less awareness of the important role of asymptomatic malaria, which may have hindered full understanding and acceptance of the study. These findings are again consistent with Okello’s study [4], which concluded that even with a good level of acceptability, a lack of understanding of the risk and role of asymptomatic malaria could contribute to issues of non-adherence. Although iMSaT communities were generally positive about Duo-Cotecxin—mostly due to its simpler dosing regimen and perceived high efficacy—the results suggest that adherence could become problematic even though general acceptability is high. While very few iMSaT participants directly mentioned their failure to finish the full dose of Duo-Cotecxin given by iMSaT teams, their responses regarding general adherence behaviour in the community suggest that it is very common to stop taking medication due to side effects or to perceptions of not being ill, and thus opting to save the drugs for later use. The study in the Zambia [3] reported similar findings, as did Okello and colleagues [4] who concluded (based on what the children’s parents suspected) that children either threw away the drugs because they feared taking them or because they did not believe they had malaria. It is also worth noting that even before the start of the iMSaT study, concerns about the drug’s affordability, availability, and side effects were very common—factors that may also influence access and adherence to the drug.

The iMSaT findings highlight the importance of educating participants about adherence and suggest that follow-up visits should continue to be an important and necessary component of iMSaT in order to ensure proper adherence to the study drug (as some respondents suggested in their recommendations). However, some of the same iMSaT participants who admitted to taking partial doses of the study drug also emphasized the importance of completing treatment as prescribed and conveyed an understanding of the dangers of not finishing the full dose. This discordance between knowledge and behaviour suggests other underlying external factors may be at play (outside the iMSaT study) such as access to treatment, cost or availability of drugs, poverty, and other structural issues.

Another finding worth highlighting is the study teams’ attitude and perseverance which participants remarked upon throughout the study. Studies of MDA in the neglected tropical diseases (including schistosomiasis, soil-transmitted helminths, and lymphatic filariasis) have found that the type of drug distributor (e.g., health worker, teacher, community-selected) and how and when they distribute are often key factors in the acceptability and uptake of MDA [25, 26]. This is a programmatic element worthy of further attention and research, especially in conjunction with perceptions of researchers and implementers, and should be incorporated into study and programme design.

### Research perceptions and community engagement

The themes which emerged during the pre-iMSaT round of qualitative data collection appear to have anticipated the barriers and challenges for household visits and highlight the need for further, targeted sensitization activities. The sensitization activities that took place may not have been as comprehensive or as effective as expected. Given the number of comments on poor sensitization (or lack thereof), and the emphasis participants’ placed on issues of blood and HIV, it is likely that some refusals or withdrawals were largely due to misinformation or misunderstanding of the purpose or intentions of iMSaT (as discussed above), or of medical research as a whole—concerns and beliefs which may have existed for many years already. Addressing these will prove challenging; fears related to blood are not easily dispelled and may actually be linked to deeper issues of power imbalance and social distance between researchers and communities [21]. Dial’s study on MDA in The Gambia [27] cited communication as the biggest challenge—specifically the gap between fieldworkers and community members, which can perpetuate such fears and rumours around blood.

Given the lack of detailed documentation with regard to how the iMSaT sensitization activities were conducted, it is difficult to know whether or not participants’ concerns were adequately addressed before the first round of iMSaT. Regardless, it is also important to recognize that these issues may not be merely a question of health education, but may require a more long-term, culturally appropriate approach to community engagement and researcher-community dialogue.

For this purpose, KEMRI-CDC has in place a community engagement strategy, which is overseen by a Community Liaison Officer. Activities include informing the community of the objectives of planned and ongoing activities, generating community acceptance and support for the programme, community mobilization for informed participation, promoting a positive image of the organization, coordinating result disseminations to community members, coordinating outreach activities, and managing rumours and propaganda arising from research activities. There is a rich set of literature exploring these elements of community engagement and offering insights from programmes which have attempted to put them into practice in various contexts [28–31]. Although post-iMSaT responses were overall more positive of KEMRI-CDC as compared to pre-iMSaT responses, one cannot know for certain whether this shift was a result of effective community engagement and sensitization activities or of the intervention itself.

### Further recommendations

The qualitative study achieved its original objectives of investigating pre- and post-implementation perceptions of screening and treatment in the absence of symptoms, and the results served to inform delivery of the iMSaT intervention. The iMSaT study (and future KEMRI-CDC research) would likely benefit from more robust, better documented community engagement activities— specifically tailored to individual studies and to the concerns of participating communities. For the remainder of the iMSaT intervention rounds, however, a short-term variation of this approach could be considered in order to open communication channels and further clarify misconceptions, thereby improving the acceptability of the study and ensuring optimum uptake and adherence to the intervention. Although community acceptance of iMSaT was generally positive, sensitization still needs to be refined and adapted to specific community concerns and misconceptions as outlined in this paper. It is imperative to have accurate, consistent, and frequent communication from researchers to community members and vice versa.

Following suggestions from community members, it is clear that future sensitization should continue to incorporate village leaders and other trusted group leaders (youth or women) who play an important role in community buy-in, as discussed in [3, 27]. These activities could consist of more interactive meetings in which community members can ask questions directly to iMSaT team members about the intervention. This would provide an opportunity to clarify the purpose of targeting and treating asymptomatic malaria, to address some participants’ expectation of drugs even after a negative test, and to reinforce the consequences of not adhering to treatment even when one does not feel sick. Regarding the dried blood spots, it should be noted that after round 1 of iMSaT, these were only collected for a subsample of the participants. Nevertheless, given the confusion surrounding the filter paper samples, an option for future studies would be to separate this from the main intervention in a more formalized way—perhaps by providing separate consent forms thereby allowing participants to opt out of the dried blood spots but still take part in the intervention. Another option would be using the RDT test cartridges as a source of blood sample for further assays and clearly explaining this to participants. Furthermore, communication channels should remain open throughout the study; information should continue to be disseminated back to the participants after the last rounds of iMSaT thereby strengthening partnership and fostering trust between researchers and communities.

The findings from this study have also raised some important questions regarding the issue of adherence and to what extent it is influenced by sensitization efforts. The data touched on perspectives from iMSaT participants who appear to understand the consequences of not adhering to treatment and yet choose to not finish their dose for various reasons. The community members who are continuously sensitized and educated yet fail to follow through illustrate the frustrating complexities of community health interventions and drug compliance. A more realistic approach may be to accept that there will always be a proportion of people who will not benefit from sensitization and may not adhere to treatment. Some may argue there is a need for further studies on this issue—one which has extensive implications for the success and impact of future public health interventions.

## Limitations

Most of the pre-implementation data were based on hypothetical scenarios of testing and treatment and thus may not have identified the full range of factors influencing participation that could occur in later rounds. They may overestimate or overemphasize the importance of negative perceptions which appear to have had little effect on actual participation given the low refusal rates in the first three rounds of iMSaT. Only six out of a total 41 intervention communities were included in the qualitative component. However, the number of interviews conducted (36 FGDs and 12 CHW interviews) comprise a fairly large sample of respondents for a qualitative study, and the data suggest that a level of data saturation was achieved as no new themes emerged in the final interviews. In addition, despite random selection of FGD participants, not all community members on the original lists were available at the time of interview. Consequently, village leaders sometimes resorted to convenience sampling, appointing available community members as replacements. Nevertheless, the study benefited from triangulation of information from several types of participants: CHWs, opinion leaders, and male and female community members of varying age, as well as from all three districts in the study area. Although data quality control strategies were implemented in an attempt to minimize potential bias from the transcription process, there are factors which might have influenced transcript content. These include translating Dholuo recordings directly into English (bypassing written Dholuo), which might have resulted in variations in facilitators’ interpretations of both local language nuances. Although the small number of communities included in this study may limit the extent to which the results are generalizable to other contexts, the findings are consistent with other studies that have examined community perceptions of research trials, in Kenya and elsewhere.

## Conclusion

This qualitative study has explored community perspectives of intermittent mass screening and treatment for malaria and has identified barriers to acceptability and implementation. The findings have implications for future mass treatment interventions as well as for wider efforts in community engagement. Further sensitization activities may help alleviate participants’ concerns by dispelling fears, clarifying misconceptions, and educating communities on the role and consequences of asymptomatic malaria (as shown in other studies). It is necessary, however, to approach community engagement and sensitization activities with realistic expectations; there will likely be a portion of community members who are not very receptive to one-off sensitization activities. A more long-term commitment to community engagement and communication may improve relations and foster trust between such community members and those conducting the research. Researchers and programmers should re-focus their attention on the growing body of information on community engagement in practice in order to build upon existing models of how research institutions and programmes can best integrate their work into study communities—thereby facilitating acceptable yet rigorous research which is more likely to have a lasting impact on the populations it serves.

## Abbreviations

CAB: community advisory board
CHW: community health worker
FGD: focus group discussion
HDSS: health and demographic surveillance system
IDI: in-depth interview
iMSaT: intermittent mass screening and treatment
IPT: intermittent preventive treatment
IRS: indoor residual spraying
ITN: insecticide-treated bed net
MDA: mass drug administration
RDT: rapid diagnostic test kits
WHO: World Health Organization
